# EjBZR1 represses fruit enlargement by binding to the *EjCYP90* promoter in loquat

**DOI:** 10.1038/s41438-021-00586-z

**Published:** 2021-07-01

**Authors:** Wenbing Su, Zikun Shao, Man Wang, Xiaoqing Gan, Xianghui Yang, Shunquan Lin

**Affiliations:** 1 State Key Laboratory for Conservation and Utilization of Subtropical Agro-Bioresources and Key Laboratory of Innovation and Utilization of Horticultural Crop Resources in South China (Ministry of Agriculture and Rural Affairs), College of Horticulture, South China Agricultural University, 510642 Guangzhou, China; 2 Fruit Research Institute, Fujian Academy of Agricultural Science, 350013 Fuzhou, China; 3 Key Laboratory of Loquat Germplasm Innovation and Utilization, Putian University, 351100 Putian, China

**Keywords:** Brassinosteroid, Transposition

## Abstract

Loquat (*Eriobotrya japonica*) is a subtropical tree that bears fruit that ripens during late spring. Fruit size is one of the dominant factors inhibiting the large-scale production of this fruit crop. To date, little is known about fruit size regulation. In this study, we first discovered that cell size is more important to fruit size than cell number in loquat and that the expression of the *EjBZR1* gene is negatively correlated with cell and fruit size. Virus-induced gene silencing (VIGS) of *EjBZR1* led to larger cells and fruits in loquat, while its overexpression reduced cell and plant size in *Arabidopsis*. Moreover, both the suppression and overexpression of *EjBZR1* inhibited the expression of brassinosteroid (BR) biosynthesis genes, especially that of *EjCYP90A*. Further experiments indicated that *EjCYP90A*, a cytochrome P450 gene, is a fruit growth activator, while EjBZR1 binds to the BRRE (CGTGTG) motif of the *EjCYP90A* promoter to repress its expression and fruit cell enlargement. Overall, our results demonstrate a possible pathway by which EjBZR1 directly targets *EjCYP90A* and thereby affects BR biosynthesis, which influences cell expansion and, consequently, fruit size. These findings help to elucidate the molecular functions of BZR1 in fruit growth and thus highlight a useful genetic improvement that can lead to increased crop yields by repressing gene expression.

## Introduction

Fruits provide humans with various nutrients, are enjoyable to eat, and are essential to our daily lives^1^. Fruit size is an important commercial trait that directly affects the quality and economic value of fruit^2^. Cultivars or lines that consistently produce large fruits are critical for grower profitability, effectively satisfy consumer demand and are preferentially selected during domestication and modern breeding processes. Though the molecular controls of the size of organs, such as leaves and flowers, are well known^3^, knowledge of fruit size regulation is unclear. For annual horticultural crops, *FW2.2* (*fruit weight 2.2*) and *FW3.2* (*fruit weight 3.2*) regulate cell division during tomato fruit size evolution^4,5^. *fw11.3* and *POS1* (*Physalis Organ Size 1*) are believed to modulate fruit size by regulating cell expansion^6,7^. In addition, a recent study demonstrated that *CsFUL1* modulates cucumber fruit elongation by modulating auxin transportation^8^. However, little is known about fruit size regulation in perennial fruit trees.

Loquat (*Eriobotrya* Lindl.) is a subtropical evergreen fruit tree belonging to the apple subfamily in Rosaceae that bears nutritious and succulent fruits. The cultivated species, *Eriobotrya japonica*, initiates bud differentiation in inflorescences in late summer, and the fruits ripen during late spring and early summer^9,10^. Under rigorous management in semiarid subtropical regions with abundant sunlight, such as the Miyi area (Panzhihua, Sichuan, China), the trees can produce fruits from December to March of the next year. An efficient cultivation system that combines various cultivars with multiple climate types has strongly improved loquat production in the last decade in China^11^. However, low yields due to small fruit and few fruit-bearing branches, as well as the short fruit shelf-life, are factors that have severely restricted the cultivation of this fruit crop^12^. Although fruit thinning^13^ and the application of plant growth regulators^14^ have been widely used to enlarge fruit size in loquat, the mechanisms of fruit size regulation have not been fully elucidated. Previously, we found that loquat proliferated only a small proportion of cell layers after fruit set and suggested that the regulation of cell size would be a more promising aim for further fruit size-related breeding^15^. Moreover, our transcriptome data revealed that a set of BR biosynthesis- and BR signaling-related genes were significantly related to cell size expansion in two hybrid lines (Supplementary Fig. 1). Nonetheless, the effects of these genes on fruit size and the pathways through which they function to regulate cell size warrant further research.

Brassinosteroids (BRs) are the sixth class of plant-specific steroidal hormones. In plants, BR biosynthesis and BR signaling genes are involved in biological processes such as cell elongation, xylem differentiation, vegetative growth, apical dominance, carotenoid accumulation and photomorphogenesis^16–22^. Similar to other plant hormones, BRs and BR signal transduction play vital roles in protecting plants from a variety of environmental stresses, such as high or low temperature, drought, and pathogen attack^23–25^. Among all BR-related genes, BZR1 (BRASSINAZOLE-RESISTANT 1) and its homolog, BES1 (BRI1-EMS-Suppressor 1), act not only as key transcription factors in the BR signaling pathway but also as hubs that integrate diverse signals to regulate plant development and environmental adaptability^26^. Although the roles that BES1^27^ and BZR1^17^ play in stem and hypocotyl elongation are well understood in *Arabidopsis*, whether and how BR synthesis or signaling genes function in fruit size development and crop yield is little known to date^28^.

In this report, we described the relationships between fruit weight and cell size in 13 loquat accessions. Then, the functions of one BR-related transcription factor, *EjBZR1*, were verified through virus-induced gene silencing (VIGS) in fruit and overexpression in *Arabidopsis*. Functional analyses revealed that larger fruits with larger cell sizes were obtained in the VIGS experiment, while a global reduction in cell size, vegetative biomass, fruit size, and petal size was shown in the overexpressing *Arabidopsis* lines. In both experiments, BR biosynthesis genes were significantly elevated/repressed, which had strong effects on cell expansion and final organ size. Further investigations revealed that *EjCYP90A*, one of the most strongly regulated BR biosynthesis genes in loquat, was an activator of cell size and organ size in both the VIGS and overexpression experiments. Furthermore, we confirmed that EjBZR1 could bind to the *EjCYP90A* promoter and suppress gene expression. This study was the first to reveal the involvement of a BR-related pathway in fruit size regulation and demonstrate the potential applicability of BR-related genes to the regulation of fruit yield and plant biomass in horticultural plants and other crops.

## Results

### Comprehensive analysis of the contribution of cell expansion to loquat fruit size

**Fig. 1:**
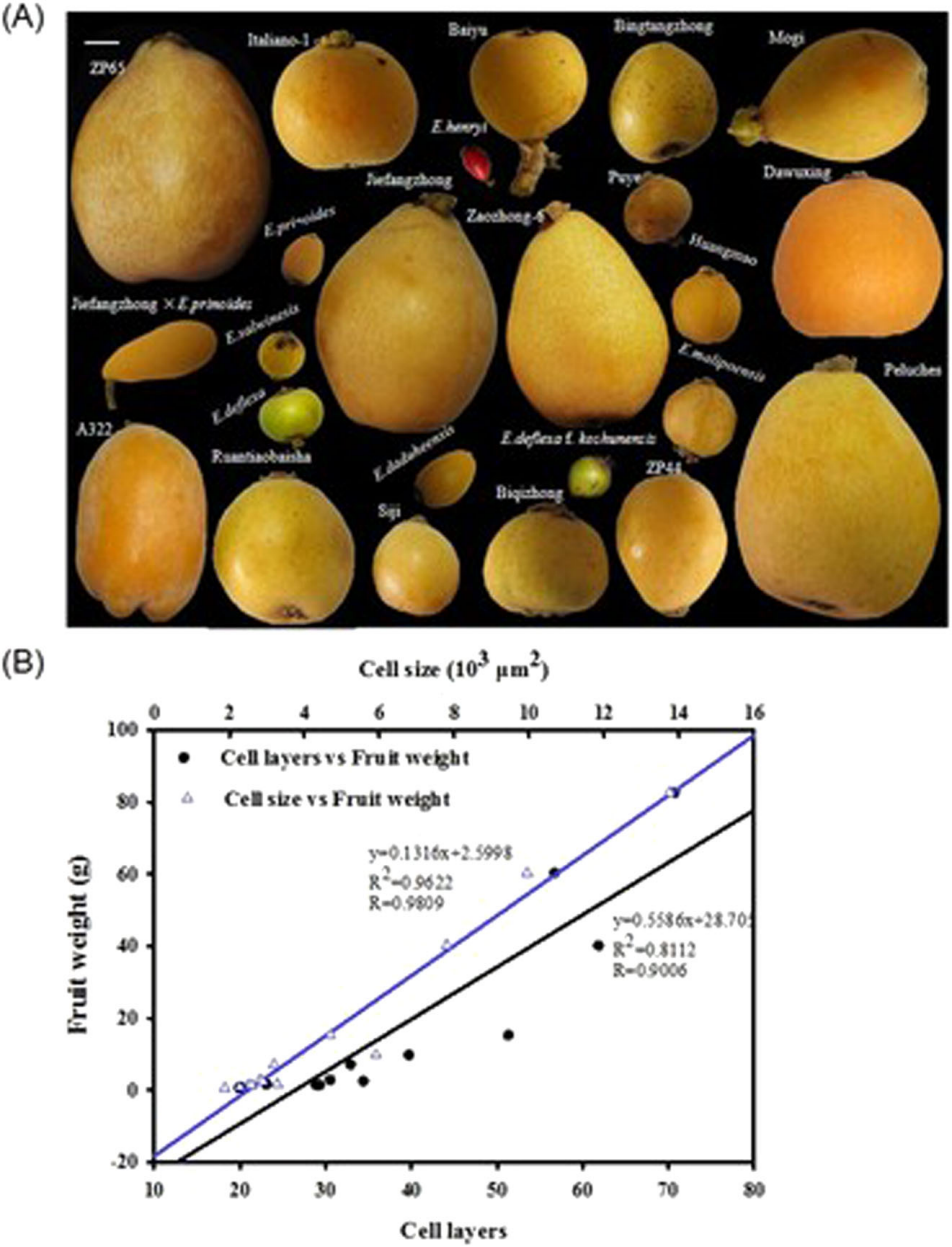
Relationships among fruit weight and fruit cell characteristics. **A** Photographs of mature fruits of various sizes, shapes, and colorations across the *Eriobotrya* genus. The bar on top left represents 1.0 cm. **B** Cell size is more strongly positively correlated than cell number with fruit weight among 13 loquat accessions. The fruit weight data are the mean values from 30 fruits of each accession. The cell layer and cell size data are the mean values from cortex sections of three representative fruits of each accession

As shown in Fig. 1A, there are ~26 species in the *Eriobotrya* genus, and their fruits have highly different sizes and shapes. However, to date, *E. japonica* is the only *Eriobotrya* species cultivated for fruit production. Small fruit size is the predominant factor impeding the cultivation of wild species. Improving the fruit size of these species would support the development of large germplasm resources for breeding this fruit (Fig. 1A). Nevertheless, the factors directly influencing the size of loquat fruits have not been determined. To understand what cellular characteristics a large loquat accession would have, a total of 13 loquat accessions were selected and comprehensively screened for factors affecting fruit size development (Supplementary Table S1).

The fruit weight of the five cultivated *E. japonica* varieties is >10.0 g (except that of the cultivated wild variety “Puye”), while the fruit weight of most wild species is <5.0 g (except that of *E. serrate*). The fruit of the cultivated line with the largest fruit (ZP65) is ~11 and 156 times heavier than those of the wild-grown “Puye” and *E. henryi*, respectively (Supplementary Table S1). This finding suggests that there is ample opportunity to increase fruit size in wild loquat and that wild species that produce fruits of similar size to those of the wild strain “Puye” might be easier to improve to produce larger fruit. The fruits of all these species were then dissected and cut into slices for cellular observation. The results showed that larger fruit size was always associated with larger fruit diameter, a thicker pericarp, more cells, and larger cells in the 13 studied accessions (Supplementary Table S1). In addition, cell size seemed to be more positively related to loquat fruit size than other factors (Fig. 1B). This finding suggests that targeting a larger cell size could be a promising approach for breeding larger fruits in loquat.

### Isolation and characterization of *EjBZR1*

**Fig. 2:**
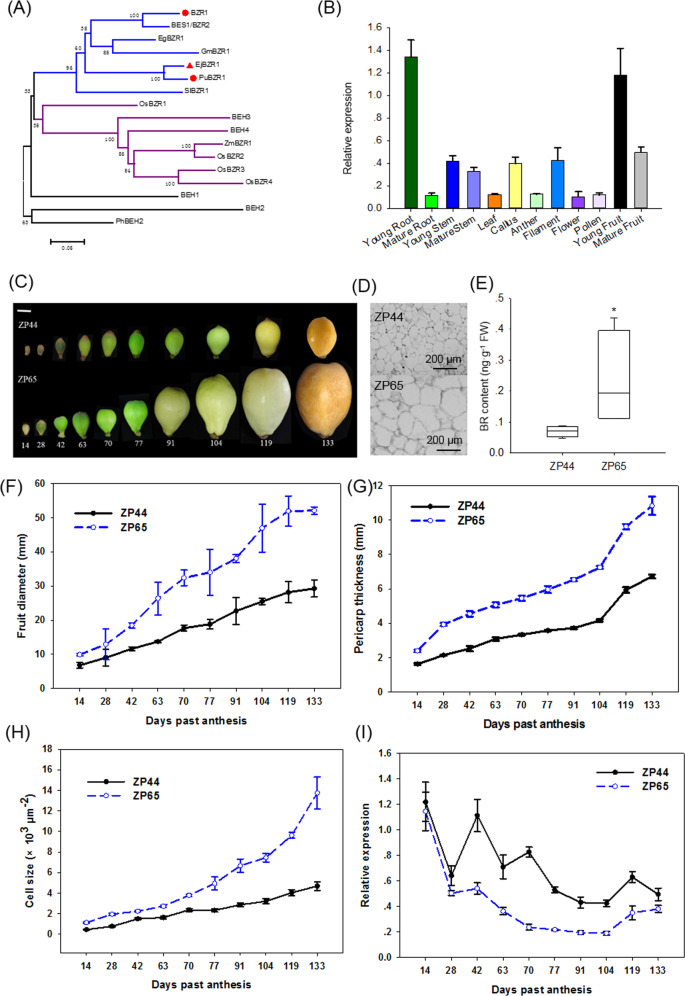
Sequence identification of *EjBZR1* and the relationship of its expression to fruit size. **A** Phylogenetic analysis of plant BZR1 family proteins. EjBZR1 clusters with PuBZR1 in the same subgroup as BZR, while OsBZRs cluster into another clade. Eg, *Eucalyptus grandis*; Ej, *Eriobotrya japonica*; Gm, *Glycine max*; Os, *Oryza sativa*; Ph, *Petunia* × *hybrid*; Pu, *Pyrus ussuriensis*; Sl, *Solanum lycopersicum;* Zm, *Zea mays*. **B** Expression patterns of EjBZR1 in diverse tissues. Error bars indicate means ± SEs (*n* = 3). **C** Fruit development of large ZP65 fruits and small ZP44 fruits, bar = 1.0 cm. **D** Fruit cortex section of the two hybrid lines. **E** Brassinolide content in ZP44 and ZP65 fruits at 42 days after anthesis. Significance testing was conducted using one-way ANOVA in SigmaPlot (*P < 0.05). Error bars indicate means ± SEs (*n* = 3). **F** Fruit diameter and **G** pericarp thickness during fruit development in the two hybrid lines. Error bars indicate means ± SEs (*n* = 15 in **F**, **G**). **H** Cell size increased during fruit size enlargement. **I** Expression of *EjBZR1* was negatively correlated with fruit size. The vertical bars represent the standard error of three replicates. (*n* = 3 in **H**, **I**)

A *BZR1/BES* homolog named *EjBZR1* was isolated from *E. japonica* cv. Zaozhong-6. This gene encodes 295 amino acids, with a calculated molecular weight of 32.25 kDa. Amino acid alignment revealed that EjBZR1 contains a DNA-binding domain with a nuclear localization signal sequence (NLS), a putative 14-3-3 binding site (RISNSAP), and a putative PEST sequence, which are typical characteristics of BZR transcription factors (Supplementary Fig. 2). In particular, the EAR (ethylene-responsive element binding factor-associated amphiphilic repression) domain (LxLxLx) was found to be localized near the C terminus of these BZR proteins, which suggests that they are potential transcription repressors. Then, a phylogenetic tree based on BZR sequences indicated that EjBZR1 closely clustered with PuBZR1 and was grouped into a clade with *Arabidopsis* BZR1 and BES1/BZR2 (Fig. 2A). Afterward, a specific tissue expression pattern for this transcription factor was detected. The data showed that *EjBZR1* was predominantly expressed in young tissues, such as young roots and young fruits (Fig. 2B). These results implied that the *EjBZR1* transcription factor might initiate its functions during early tissue development.

### *EjBZR1* expression is negatively correlated with cell size and fruit enlargement

To study the possible role that EjBZR1 plays during fruit enlargement, the correlation of the *EjBZR1* gene expression pattern with fruit growth was analyzed using the fruits of hybrid lines derived from “Zaozhong-6” (ZP44 and ZP65, Fig. 2C) maintained by our laboratory. The fruit weight of these lines distinguishes them from each other; ZP44 set fruits of 15.21 g weight, while single fruits of ZP65 weighed as much as 82.69 g (Supplementary Table S1), and cell observation showed that cells of ZP65 fruits were significantly larger than those in ZP44 (Fig. 2D). The brassinolide content in large-sized ZP65 fruits was more than three times higher than that in small-sized ZP44 fruits (Fig. 2E). Developmental comparisons of these lines during the 10 phases of fruit development showed that fruit size acutely increased in both fruit diameter and pericarp thickness ~77 days after blooming (Fig. 2F, G). Consistent with the diameter enlargement and pericarp thickening, the fruit flesh cells markedly enlarged from 70-77 days after bloom (Fig. 2H). The obvious increase in cell size was accompanied by a larger gap in fruit size between ZP44 and ZP65. Gene expression assays showed that the expression levels of *EjBZR1* declined as fruit size increased in both lines, and the opposite pattern was observed for cell expansion (Fig. 2I). Moreover, the expression level of *EjBZR1* in ZP44 (small fruits) was always higher than that in ZP65. All these data indicate that *EjBZR1* might be a negative regulator of fruit size in loquat.

### Suppressing *EjBZR1* expression promotes fruit enlargement

**Fig. 3:**
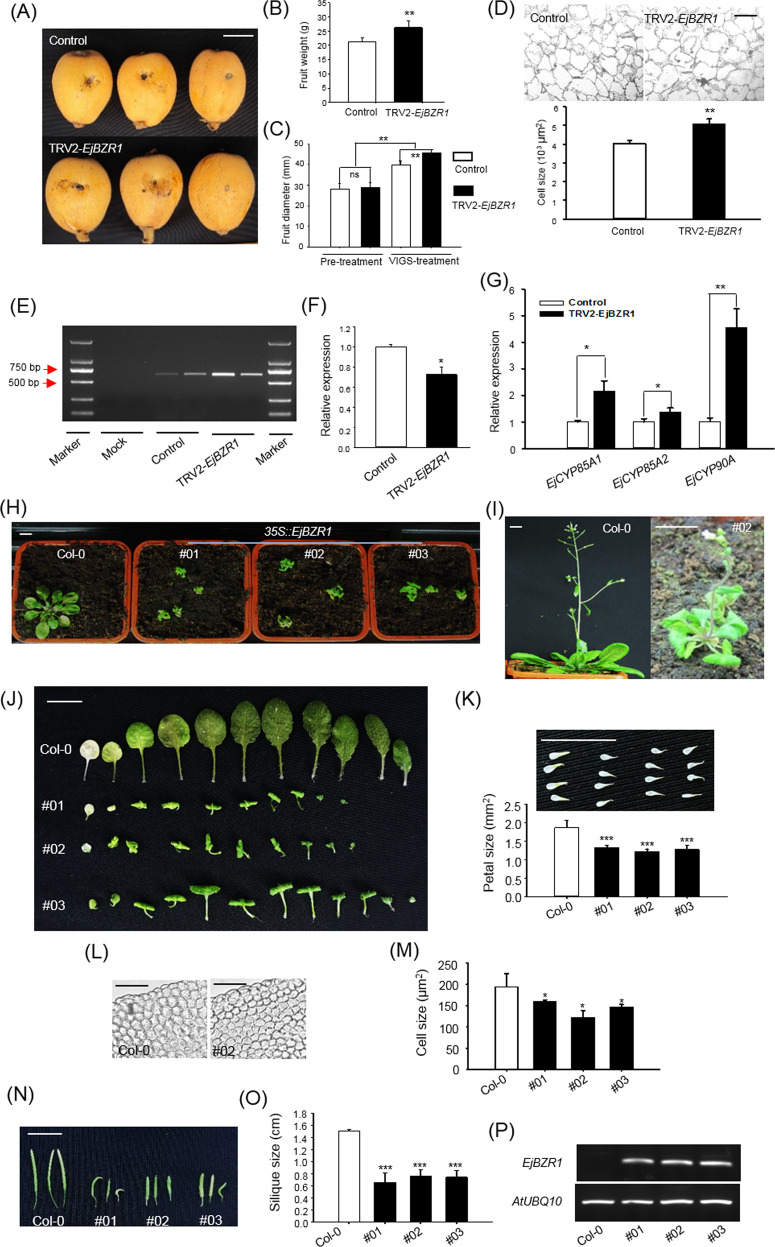
*EjBZR1* functions as a repressor of fruit and organ size development. **A** VIGS of *EjBZR1* promoted fruit growth. Bars represent 1.0 cm. VIGS treatment increased fruit weight (**B**) as well as fruit diameter (**C**) and cell size (**D**). Bars = 100 μm in **D**. Error bars indicate means ± SEs (*n* = 20 in **B**, **C** and *n* = 3 in **D**). **E** Amplification of the 615-bp-length coat protein cDNA confirmed a working VIGS system in the treated fruits. **F** VIGS reduced *EjBZR1* expression in loquat fruit. **G** Reducing the *EjBZR1* expression level promoted BR biosynthesis gene expression in VIGS-treated fruits. The vertical bars represent the standard error of triplicate experiments. *P < 0.05 and **P < 0.01 by Student’s *t*-test. Ectopic expression of *EjBZR1* decreased plant size in *Arabidopsis* (**H**, **I**). The plants shown in **H** were 28 days after germination. **J** Extremely small and curly rosette leaves in the transgenic lines. **K** Smaller petals in the transgenic lines. **L** Epithelial petal cells of Col-0 and line 2. **M** Petal cell size decreased in all overexpression lines. **N**, **O** Smaller and abnormal siliques in the OE lines. **P** Semiquantitative RT-PCR of *EjBZR1* in overexpression lines. Significance testing was conducted using one-way ANOVA in SigmaPlot (*P < 0.05, **P < 0.01). Bars = 1.0 cm in **H**–**K** and **N**, and bar = 50 μm in **E**. Error bars indicate means ± SEs (*n* = 3) in **F**, **G**, **K**, **M**, and **O**

To verify the hypothesis that *EjBZR1* is a negative regulator of fruit size, VIGS was first performed on “Zaozhong-6” fruit ~56 days after anthesis; this is when fruits initiate cell size expansion, according to our previous research^15^. After ripening, the gene-silenced fruits were discovered to be larger than the fruits injected with an empty vector (Fig. 3A), with their fruit weight being ~24% higher than that of the control fruits (Fig. 3B). Histological observations of the fruit cortex revealed that the cells in the VIGS fruits were significantly larger than those in the control fruits (Fig. 3C, D).

To ensure that the changes in fruit size were caused by the reduction in *EjBZR1* abundance due to the VIGS treatment, the coat protein coding sequence carried by the TRV2 vector was first amplified. The results showed that 615-bp products were obtained in both the VIGS (injected with TRV1 + TRV2-*EjBZR1*) and control (injected with TRV1 + TRV2-Empty) fruits, while no amplification product was obtained in the mock fruit (without vector injection) with the specific primer pair (Fig. 3E). PCR amplification confirmed that the vectors were effective after being injected into loquat fruit and suggested that the VIGS system worked for the loquat fruits in our study. Relative quantitative data revealed that the *EjBZR1* expression level was reduced by ~30% in the VIGS-treated fruits (Fig. 3F), while the expression levels of BR biosynthesis rate-limiting genes, especially that of *EjCYP90*A, were notably elevated (Fig. 3G and Supplementary Fig. S3).

### Overexpression of *EjBZR1* suppresses *Arabidopsis* plant size

To further confirm that *EjBZR1* acts as a repressor of organ size, a plant binary expression vector originating from pBI121 was constructed for plant genetic transformation in *Arabidopsis*. A total of six overexpression lines were obtained in our transgenic experiment. The plant size of all the overexpressed lines was severely reduced and was significantly smaller than that of wild-type Col-0 in the T3 generation at 30 days after germination (Fig. 3H). More precisely, the plant height (Fig. 3I and Supplementary Fig. S4A), leaf size (Fig. 3J), petal size (Fig. 3K and Supplementary Fig. S4B), and fruit size (Fig. 3N) of the genetically modified lines were all markedly smaller than those of the nontransgenic wild-type plants, and the leaves of the transgenic plants were severely curled (Fig. 3J).

Cellular observation and measurements of the petals of the first terminal flowers showed that the cell size of the overexpression lines was significantly smaller than that of Col-0 (Fig. 3L, M) and resulted in at least 29.0% smaller petals in the overexpression lines (Supplementary Fig. S4B). Semiquantitative RT-PCR confirmed that all transgenic lines transcribed abundant *EjBZR1* RNA, while no *EjBZR1* transcription was detected in the wild plant (Fig. 3O). The relative quantitative data revealed that the expression of certain genes, especially the BR biosynthesis rate-limiting *CYP90*s and *CYP85*s, was reduced by several times and even dozens of times in the overexpression lines (Supplementary Fig. S5). Together with the results from the gene expression experiment in VIGS loquat fruit, these results suggest that the repression of BR biosynthesis gene expression is a crucial means by which EjBZR1 regulates fruit size.

### *EjCYP90A* functions as an activator of fruit growth in loquat

**Fig. 4:**
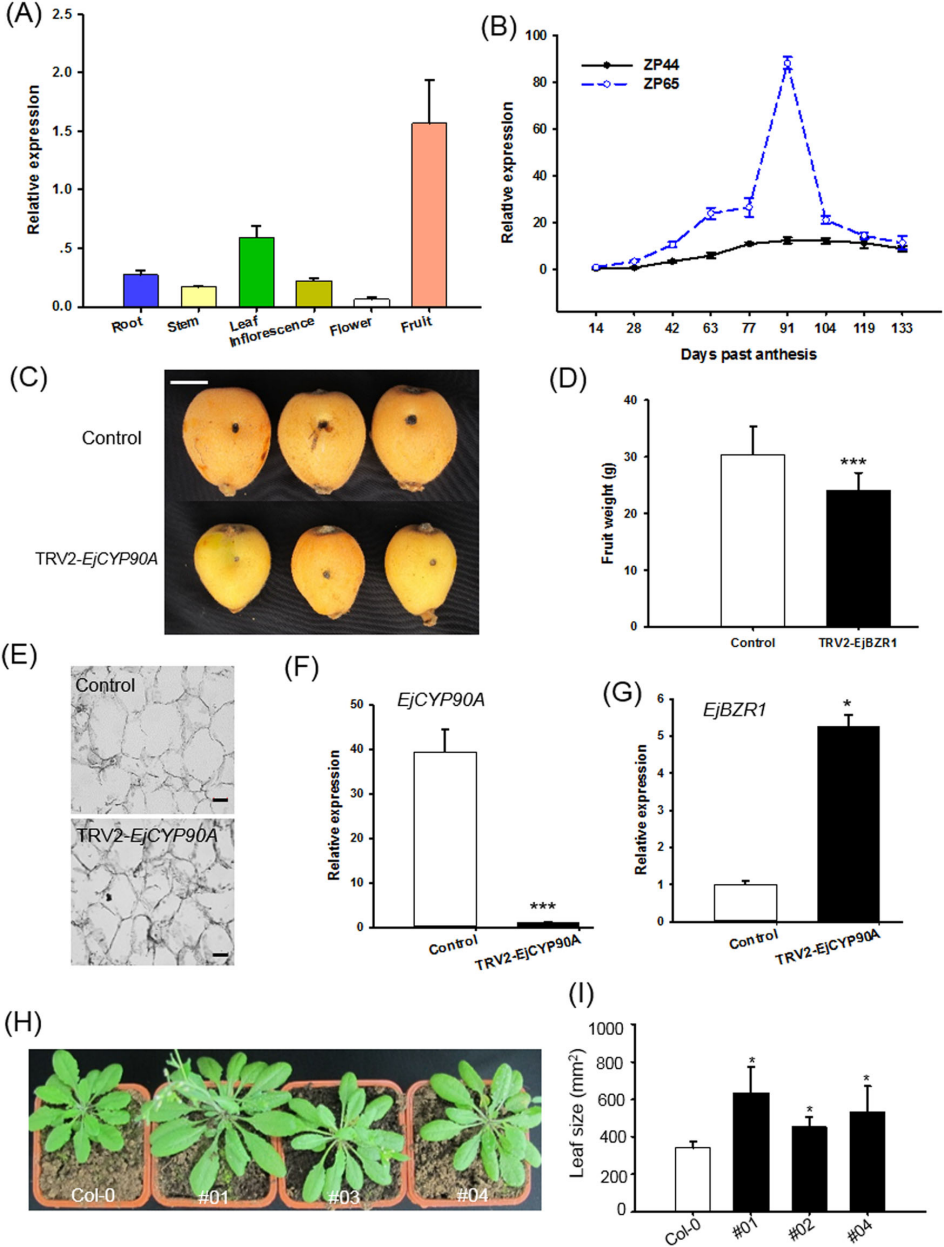
Functions of *EjCYP90A* in fruit growth. **A** Specific expression pattern of *EjCYP90A* in different tissues. **B** Comparisons of *EjCYP90A* expression patterns in ZP44 and ZP65 fruits. Error bars present the means ± SEs, with three biologically independent samples for expression detection in **A** and **B**. **C** Fruits under VIGS treatment for *EjCYP90A*. Bars = 1.0 cm. **D** Reduction in *EjCYP90A* expression decreased fruit weight. Error bars indicate means ± SEs (*n* = 20) in **D**. **E** Flesh sections of TRV2-*EjCYP90A*. Bars = 50 μm. **F** VIGS treatment significantly reduced *EjCYP90A* transcript abundance. **G** The repression of *EjCYP90A* transcript abundance induced higher *EjBZR1* expression levels. Significance testing was conducted using one-way ANOVA in SigmaPlot (*P < 0.05, ***P < 0.001). Bars = 50 μm. **H** Overexpression of *EjCYP90A* promoted *Arabidopsis* plant growth. **I** Larger leaf size in *EjCYP90A* overexpression lines. Error bars indicate means ± SEs (*n* = 3) in **F**, **G**, and **I**

*EjCYP90A* was one of the most strongly elevated BR biosynthesis-related genes in *EjBZR1-*silenced fruits (Fig. 3G). Similarly, the expression levels of *CYP90*-homologous genes were significantly repressed in the *EjBZR1* overexpression lines (Supplementary Fig. S5). Based on these results, *EjCYP90A* is suggested to be one of the most important targets of EjBZR1, and its role in fruit growth is of interest. The coding sequence of *EjCYP90A* was then cloned from “Zaozhong-6” fruit, as was performed for *EjBZR1* (Supplementary Fig. S6). The tissue expression data showed that *EjCYP90A* was predominantly expressed during the rapid expansion phase of fruit development (Fig. 4A). More importantly, *EjCYP90A* showed distinctly higher expression levels in ZP65 fruits than in ZP44 fruits during the fruit size expansion phase (Fig. 4B), especially during the phases in which fruit cells expand markedly, as shown in our former study^15^.

Consequently, VIGS and ectopic transformation were performed to reveal the functions of *EjCYP90A* in fruit development. First, the silencing of *EjCYP90A* resulted in small fruit (Fig. 4C), i.e., a fruit weight reduction of ~20.91% compared with the control (Fig. 4D), and smaller cell size (Fig. 4E). Then, quantitative gene expression assays showed that *EjCYP90A* expression was markedly reduced in VIGS-treated fruits (Fig. 4F); in these fruits, the transcript abundance of *EjBZR1* was acutely elevated (Fig. 4G). Furthermore, the overexpression of this gene in Col-0 *Arabidopsis* generated larger plants (Fig. 4H, I). Taken together, these results indicate that *EjCYP90A* is an activator of fruit growth in loquat.

### EjBZR1 directly binds to the *EjCYP90A* promoter to repress gene expression

**Fig. 5:**
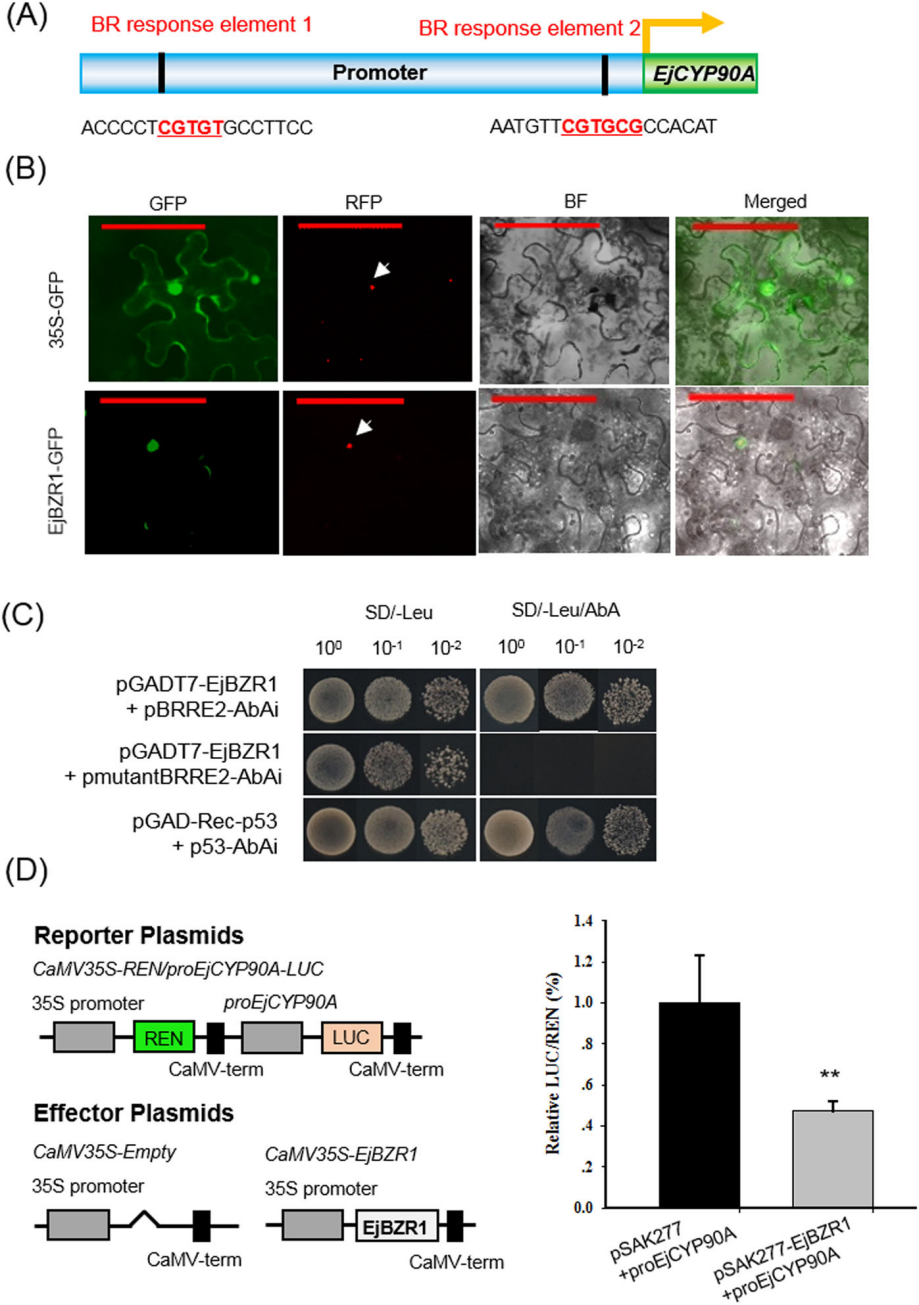
Direct targeting of *EjCYP90A* by EjBZR1. **A** Schematic representation showing the localization of the putative binding sites of EjBZR1 in the *EjCYP90A* promoter. **B** Subcellular localization of EjBZR1. The 35S::GFP and 35S::EjBZR1-GFP plasmids were transformed into *N. benthamiana* cells. Red fluorescence signals were used as nuclear markers. Scale bars = 100 μm. **C** Yeast one-hybrid assays of EjBZR1 binding to the *EjCYP90A* promoter. The constructs pGAD-Rec-p53 and p53-AbAi were used as positive controls, and pGADT7-EjBZR1 and pAbAi-mBRRE2 were used as negative controls. Three independent replicates were carried out in **B** and **C**. **D** EjBZR1 represses *EjCYP90A* expression in *N. benthamiana* leaves through the dual-luciferase (LUC) system. Relative Renilla (REN) luciferase activity was used as an internal control. The data are presented as means ± standard error (*n* = 6). **P < 0.01

The *EjCYP90A* promoter sequence (1906 bp) was isolated from “Zaozhong-6”. Sequence analysis demonstrated that there were two distinct brassinosteroid response elements (BRRE, CGTGT/CG) upstream of *EjCYP90A*, −178 to −183 bp (CGTGTG) and −1717 to −1722 bp (CGTGCG) (Fig. 5A and Supplementary Fig. S7). Subcellular assays demonstrated that EjBZR1 proteins were localized predominantly in the nucleus (Fig. 5B), which supports the hypothesis that EjBZR1 is a transcription factor and may directly regulate the expression of BR biosynthesis genes.

A previous study revealed that BZR1 can bind to the BRREs (CGTGT/CG) of BR biosynthesis genes to repress BR biosynthesis^17^. To examine the ability of EjBZR1 to bind to the BRREs in the *EjCYP90A* promoter, yeast one-hybrid (Y1H) assays were carried out. The pGADT7-EjBZR1 and pAbAi-BRRE2 cotransformants but not the pGADT7-EjBZR1 and pAbAi-mBRRE2 transformants grew well on SD/-Leu medium with AbA (aureobasidin A), suggesting that EjBZR1 specifically binds to the binding site of the *EjCYP90A* promoter (Fig. 5C). To further determine whether EjBZR1 directly associates with the promoter of *EjCYP90A* in vivo, dual-luciferase assays were carried out. Coexpression assays of the *EjCYP90A* promoter and EjBZR1 showed that EjBZR1 significantly suppresses the transcriptional activity of *EjCYP90A* compared with that in the cotransformation of the empty vector with the *EjCYP90A* promoter (Fig. 5D). Taken together, these findings confirmed that the EjBZR1 transcription factor negatively regulates the expression of *EjCYP90A* by directly binding to the BRRE of its promoter.

## Discussion

This study revealed the correlations of cell number (cell layers) and cell size (cell area) with fruit weight as well as with fruit diameter and pericarp thickness in 13 loquat accessions, including five *E. japonica* accessions (the cultivated species) and eight wild relative species (Fig. 1 and Supplementary Table S1). Previous genetic studies on tomato, the model plant for fruit research, suggested high impacts of cell division on fruit size evolution^4,5^. However, our data demonstrated that both cell division and cell size were involved in fruit size during the evolution of the *Eriobotrya* genus but that cell size is predominantly responsible for fruit enlargement during the development of this fruit crop. This finding is inconsistent with findings from relatives of this crop, such as apple^29^, pear^30^, and plum^31^; in these fruits, cell number is believed to be a more important factor than cell size for controlling fruit size. Cell proliferation may be one of the most important factors leading to the fruit size difference among these species, as the cell layers of all these fruits proliferate after anthesis, and fruit size is strongly and positively correlated with the number of cell layers. However, our previous study demonstrated that loquat fruits possess weak cell division ability and that only approximately one-third of the pericarp cell layers proliferate after blooming^15^. In contrast, cellular studies of tomato lines^32^ and grapes^33^ support our view that cell size contributes greatly to fruit size. This finding suggests that there might be unique size evolution patterns among different crops or populations and even among close relatives, such as loquat and apple. Furthermore, our study indicates that cell size is a promising target for fruit size breeding in loquat.

Previous reports demonstrated that CYP90s possess C-3, C-22 or C-23 hydroxylation abilities and play vital roles in the catalysis of early BR intermediates in *Arabidopsis* to promote plant growth^34–37^. Mutations of *CYP90A* (also named *CPD*, *CONSTITUTIVE PHOTOMORPHOGENESIS AND DWARFISM*) alleles in *Arabidopsis*^19^ and rice^38^ led to a severe dwarf plant phenotype due to a deficiency in BR accumulation. In contrast, the overexpression of *PeCPD* (*Populus euphratica*) in *cpd Arabidopsis* plants restored the phenotype to that of the wild type^39^. *CYP90A* is well known to play a crucial role in *Arabidopsis* cell elongation, which regulates plant and organ size^19^. EjCYP90A shares notably high amino acid sequence identity with *Arabidopsis* CYP90A and *Populus* PeCYP90A and contains all the conserved domains of CYP90s^19,39^, suggesting its possible function as a putative CYP90A in loquat. In addition, tissue-specific and fruit developmental gene expression data in our study revealed that the expression level of *EjCYP90* was strongly and positively correlated with cell expansion and fruit size in loquat (Fig. 4A, B). To confirm whether loquat *EjCYP90A* has similar functions to *AtCYP90A* in cell size and plant organ growth regulation, we performed VIGS on loquat fruit and transgenic experiments in Col-0 *Arabidopsis*. The fruit VIGS treatment resulted in smaller cells and fruits (Fig. 4C–G), while overexpression considerably promoted plant growth in Col-0 (Fig. 4H, I). The results were similar to those from the overexpression of *Populus* BR biosynthesis genes, *PeCPD* and *PtCYP85A3*, which promoted *Arabidopsis* plant growth^39^. Taken together, the results of the experiments on *EjCYP90A* indicated that it functions similarly to CYP90A1/CPD and is a positive regulator of cell growth and fruit size in loquat.

**Fig. 6:**
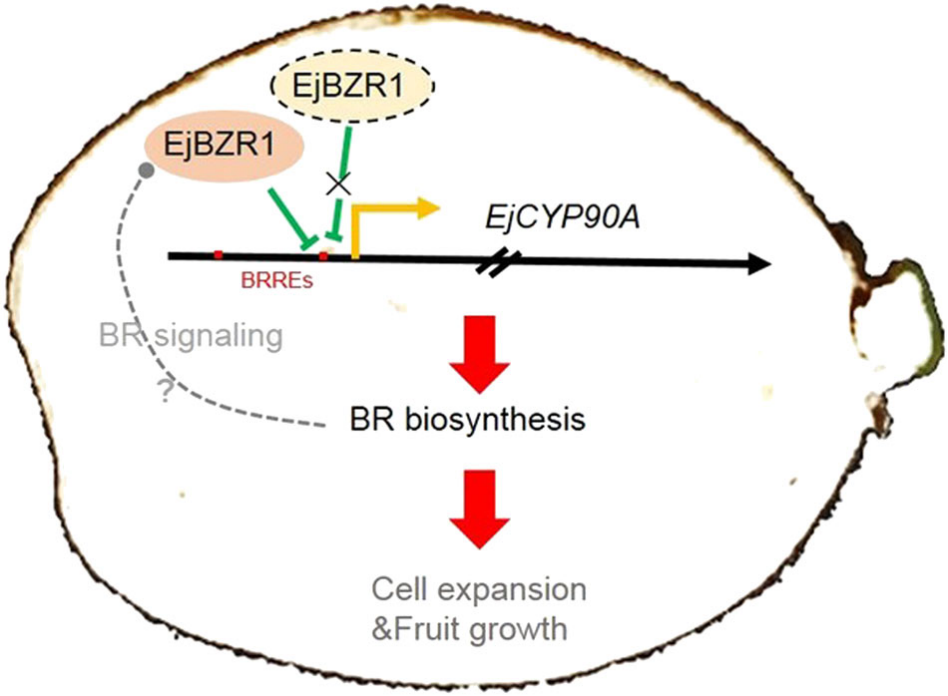
Proposed model explaining the function of EjBZR1 in regulating cell size and fruit growth in loquat. EjBZR1 is thought to downregulate the expression of *EjCYP90A* by directly binding to the BR response element (CGTGTG) of the *EjCYP90A* promoter to influence BR biosynthesis. Meanwhile, the reduction in *EjCYP90A* feedback regulates the expression of *EjBZR1* through an unknown pathway. Lowering the abundance of the EjBZR1 transcript greatly elevates the expression of *EjCYP90A*, increasing BR biosynthesis, thereby promoting cell expansion and resulting in larger fruit. T-lines indicate repressive action, arrows indicate induction, and the dashed line indicates the putative signaling pathway

In this study, we showed for the first time that EjBZR1 is a repressor of cell expansion and fruit growth (Fig. 3). In *Arabidopsi*s, it is known that nuclear-localized BZR1 is a transcriptional repressor that binds directly to the promoters of BR biosynthetic genes^17,40^. The transcriptional repression ability is believed to be correlated with the EAR domain in the C termini of BZR1/BES1 proteins. The EAR domain can directly bind to BRRE elements and recruit corepressors such as MYBL2 (MYELOBLASTOSIS FAMILY TRANSCRIPTION FACTOR-LIKE 2), HAT1 (HOMEOBOX ARABIDOPSIS THALIANA 1)^41^, TPL (TOPLESS)^42^ and HDA19 (HISTONE DEACETYLASE 19)^43^ to BRREs to form corepressor complexes and suppress the expression of BR biosynthesis genes, such as *CPD*^17^. Sequence alignment showed that EjBZR1 shares a high amino acid sequence identity with BZR1/BES1 and that there is also an EAR domain in the C terminus of EjBZR1 (Supplementary Fig. S2). Meanwhile, *EjCYP90A* (an ortholog of *CPD*) was one of the most highly elevated genes after the *EjBZR1*-VIGS treatment in loquat fruit (Fig. 4G). Consistent with those in the BZR1 overexpression plants, the expression levels of *CPD* and its homologs in the *EjBZR1* transgenic plants were significantly repressed (Supplementary Fig. S4). As in the regulatory sequence of *CPD*^17^, two BRREs were discovered in the *EjCYP90A* promoter (Supplementary Fig. S7), and nuclear-localized EjBZR1 was able to bind to BRRE2 and repress *EjCYP90A* transcription (Fig. 5C). It remains unknown whether EjBZR1 recruits corepressors to BRRE upstream BR biosynthesis genes, such as EjCYP90A, as BZR1 does. However, a simple model was established here to explain the mechanism through which EjBZR1 regulates cell size and fruit growth in loquat (Fig. 6). That is, EjBZR1 downregulates *EjCYP90A* expression by directly binding to the BR response element (CGTGTG) in the proximal *EjCYP90A* promoter region to modulate BR biosynthesis. The reduction in *EjBZR1* transcript abundance is accompanied by greatly elevated *EjCYP90A* expression and increased BR biosynthesis, thereby promoting cell expansion and resulting in larger fruit (Fig. 6).

## Materials and methods

### Plant materials and growth conditions

As shown in Supplementary Table 1, a total of 13 loquat accessions (8 wild species and 5 cultivated lines) were investigated in this study. All trees were cultivated under regular management conditions in the loquat germplasm resource preservation garden (South China Agricultural University, Guangzhou, China). Thirty mature fruits of each accession were used to measure fruit weight and size, while flesh samples of 3 fruits of each accession were fixed for histological analyses. Fruits from 10 growth phases of two Zaozhong-6 hybrids, ZP44 and ZP65 (which have extremely small and large fruit sizes, respectively), were used for gene expression assays during the fruit growth season from November 2017 to April 2018. The growth phases for the ZP44 and ZP65 fruit samples were established according to our previous study of Zaozhong-6^15^ and are shown in Fig. 2. Samples from these growth phases were collected to identify the transcriptional changes in *EjBZR1* and cellular development during fruit growth. The fruit diameter and cortex thickness of 15 fruits of the two lines were measured at each developmental stage. Roots, stems, mature leaves, calli, inflorescences, flowers (dissected into anthers, filaments, and pollen for *EjBZR1*), young fruits (42 days after anthesis), and mature fruits (133 days after anthesis) were collected for gene expression assays. Callus was previously induced by our lab, and the calli were conserved in MS medium with 1.0 mg L^−1^ 6-BA and 0.1 mg L^−1^ NAA at pH = 6.0.

*Arabidopsis* and *Nicotiana benthamiana* were used for stable and transient genetic transformation, respectively. Transgenic plants were grown in a greenhouse under long-day conditions (16 h light/8 h dark) at 22 °C. Plants from 4-week-old T3 *Arabidopsis* overexpression lines were collected for gene expression assays.

### BR content assays

One hundred milligrams of fruit flesh tissue from each sample taken 42 days after anthesis was ground into a fine powder with tetragonal zirconium polycrystalline and used for BR extraction. Then, 1.0 mL of 50% acetonitrile (acetonitrile:H_2_O = 1:1) was added to each extraction and incubated on ice for 4 h. The extraction tube was centrifuged at 12,000 rpm and 4 °C for 10 min. Then, double-layered solid phase extraction (DL/SPE) combined with boronate affinity polymer monolith microextraction (BA/PMME) was used to purify BR and related hormones. Finally, the BR contents of the samples were determined by a Thermo Scientific™ Vanquish™ UHPLC system (Waters, USA) according to previously described methods^44^. A brassinolide calibration curve (*Y* = 7.116e4X, *R*^2^ = 0.9992) was obtained using brassinolide (CAS: 72962-43-7) at concentrations ranging from 0.2 to 300 ng mL^−1^ (0.2, 0.5, 1, 10, 40, 80, 120, 160, 200, and 300 ng ml^-1^) purchased from Sigma-Aldrich. For each line, the BR levels of three biological replicates were detected.

### Fruit size-regulating gene selection

Fruits from the ZP44 and ZP65 lines at 14, 42, 77, 104, and 133 days after anthesis were subjected to RNA-seq and analyzed in our former work^45^. The transcriptome data are available at https://www.ncbi.nlm.nih.gov/sra/PRJNA721113. Genes of interest, including *EjBZR1*, shown in Supplementary Fig. 1, were selected as candidate targets for further study in this work.

### Nucleic acid extraction, gene isolation, and sequence analyses

Total RNA was prepared using the EASYspin Plus plant RNA extraction kit (Aidlab, China) according to the manufacturers’ protocols, and the PrimeScript^TM^ RT reagent Kit (TaKaRa, Japan) was used to synthesize the first-strand cDNA of the plant samples. Young “Zaozhong-6” leaves were collected for genomic DNA extraction according to the M5 CTAB plant gDNA extraction kit (Mei5 Biotechnology Co., Ltd, Beijing, China) user protocol.

Fruit cDNA was used for *EjBZR1* and *EjCYP90A* coding region amplifications with the following primer pairs: *BZR1-F:* 5′-ATGACGTCTGATGGGGC-3′, *BZR1-R:* 5′-TTAAATCCGAGCCTTTCCATTC-3′, and *CYP90-F:* 5′-ATGGATTTCCTCTT CTCG-3′ *CYP90-R:* 5′-TTACTCTTTACATTGCCCAC-3′. Multiple sequence alignment was performed using ClustalX (http://www.ebi.ac.uk) and MEGA5^46^. The promoter sequence of *EjCYP90A* was amplified with DNA from young “Zaozhong-6” leaves, and BZR1 protein binding sites of *proEjCYP90A* were analyzed on PLACE (https://www.dna.affrc.go.jp/PLACE/?action=newplace).

### *Arabidopsis* transformation of EjBZR1 and EjCYP90A and VIGS in loquat fruit

*EjBZR1 and EjCYP90A* were cloned into the pBI121 vector to construct the *35* *S::EjBZR1* and *35* *S::EjCYP90A* vectors for genetic transformation in *Arabidopsis*. The overexpression vectors were transferred into Col-0 *Arabidopsis* via the floral-dip method as previously described^47^. Conserved *BZR1* and *CYP90* sequences were identified, amplified, and fused into the TRV2 vector to inhibit the expression of *EjBZR1* or *EjCYP90A* in loquat fruit. VIGS with *EjBZR1* or *EjCYP90A* on loquat fruits was performed as described previously for cherry^48^. TRV2-empty, TRV2-EjBZR1, and TRV1 vectors were introduced into *Agrobacterium tumefaciens* strain EHA105. The strains harboring expression constructs were freshly grown on lysogeny broth (LB) medium with antibiotic selection (50 μg mL^–1^ kanamycin, 50 μg mL^–1^ rifamycin, and 50 μg mL^–1^ streptomycin) and incubated at 28 °C at 200 rpm for ~16 h, until reaching OD600 = 2. The EHA105 cells were pelleted by centrifugation at 4500×*g* for 10 min, and the supernatants were discarded. The pellets were then resuspended in freshly made MMA buffer (10 mM MgCl_2_, 10 mM MES/KOH pH 5.6, 150 μM acetosyringone) and diluted to OD600 = 0.4. Twenty fruit clusters in the same growth stage on the south side of the Zaozhong-6 tree canopy were selected for VIGS treatment; from each cluster, four fruits were selected. Twenty microliters of TRV2-empty + TRV1 and TRV2-EjBZR1 + TRV1 mixed *Agrobacterium* cells were infiltrated with an Injex-30 injector (INJEX, Germany) into each fruit near the equator at 56 days after anthesis on January 2nd, 2019 (the VIGS of EjCYP90A was carried out at 77 days after anthesis). The treated clusters were bagged soon after injection to elevate the surrounding air humidity. For the EjBZR1-VIGS treatment, fruits ripened 5 weeks after injection (approximately four weeks for the EjCYP90A-VIGS treatment). Twenty mature fruits from each treatment were used for ultimate fruit size detection, and three of them were used for cortex section preparation.

All nucleotide sequences were amplified with PrimeSTAR® HS DNA Polymerase (TaKaRa, Japan) and fused into linearized vectors with an In-Fusion HD Cloning Kit (Clontech, USA). Detailed sequences of all the primer pairs used for vector construction are listed in Supplementary Table S2.

### Transient expression assays

The promoter sequence of *EjCYP90A* was amplified and fused into the pGreen-0800 vector to construct the reporter vector, while the coding region of *EjBZR1* was fused into the pSAK277 vector to construct the effector vector^49^. The primer sequences are listed in Supplementary Table S2. The effector and reporter vectors were then transformed into *Agrobacterium tumefaciens* EHA105. Then, the mixed *Agrobacterium* solution with effector and reporter vectors was injected into *N. benthamiana* leaves as previously described^49^. Firefly and Renilla luciferase activities were assayed using a Dual-Luciferase Reporter Assay kit (Promega, America) according to the manufacturer’s protocols. The coding region of *EjBZR1* was fused into pGreen-35S-green fluorescent protein (GFP) and injected into *N. benthamiana* leaves as performed in luciferase assays. Images of EjBZR1-GFP AND 35S-GFP were captured via an Observer D1 fluorescence microscope system (Carl Zeiss, Germany). For the subcellular localization, three biological replicates were carried out for each injection. For the luciferase activity assays, six biological replicates were carried out for each injection.

### Yeast one-hybrid assays

Yeast one-hybrid (Y1H) assays were performed according to the Matchmaker Gold Y1H system user manual (Clontech, America) to detect the binding of EjBZR1 to the *EjCYP90A* promoter. The *EjCYP90A* promoter contains two putative BZR1 binding elements (BRREs, CGTGT/CG). The BRRE in the proximal promoter region was inserted into the reporter vector pAbAi as bait. Meanwhile, the CDS of *EjBZR1* was fused to the pGADT7 vector. Then, recombinant pGADT7-EjBZR1 was transformed into the Y1HGold yeast strain with the linearized reporter plasmid pAbAi-BRRE2 or pAbAi-mBRRE2 (mutant motif) to determine the protein–DNA interactions. Three biological replicates were carried out for each combination. The primers used in this assay are listed in Supplementary Table S2.

### Quantitative real-time PCR assays

The expression levels of the genes were analyzed using quantitative real-time PCR. The gene-specific primers of loquat were designed using the BatchPrimer3 program^50^, and eight brassinosteroid biosynthesis-related genes in *Arabidopsis*^51^ were selected to demonstrate that the expression of EjBZR1 regulates brassinosteroid biosynthesis. The gene expression patterns of three *CYP450* family genes, including *EjCYP85A1/2* and *EjCYP90A*, were also detected in *EjBZR1*-silenced loquat fruit. Our previously selected *EjRPL18* (MH196507)^52^ was used as the reference gene for the loquat fruit gene expression assays, and *AtUBQ10* (AL161503) was used for *Arabidopsis*. For each stage or treatment, three biological samples were subjected to gene expression assays. Detailed primer sequence information is provided in Supplementary Table S3. All biological samples were measured in triplicate and analyzed in a LightCycler 480 (Roche) using iTaq^TM^ universal SYBR Green Supermix (Bio-Rad, USA).

### Histological analysis

Three representative fruit samples from diverse loquat accessions, developmental phases, and treatments were fixed in formalin-acetic acid-alcohol (FAA) solution for at least 48 h. Cross-sections were obtained according to previously described methods^15^. Then, the cortex cells of each section were observed under a light microscope with Axio Vision LE64 software (Carl Zeiss, Germany). To determine the effects of *EjBZR1* on the cell size of *Arabidopsis*, petals of newly opened flowers were observed directly. The cell size in each sample was obtained using Image-Pro Plus 6 software (Media Cybernetics, America).

## Supplementary information

EjBZR1 supplementary -revised
