# Supplementary material for: EjBZR1 represses fruit enlargement by binding to the EjCYP90 promoter in loquat
Source: Hortic Res. 2021 Jul 1;8:152. doi: 10.1038/s41438-021-00586-z (PMC8245498; doi:10.1038/s41438-021-00586-z)
Supplement: Supplementary file 1 — EjBZR1 supplementary -revised [file 41438_2021_586_MOESM1_ESM.docx]

**
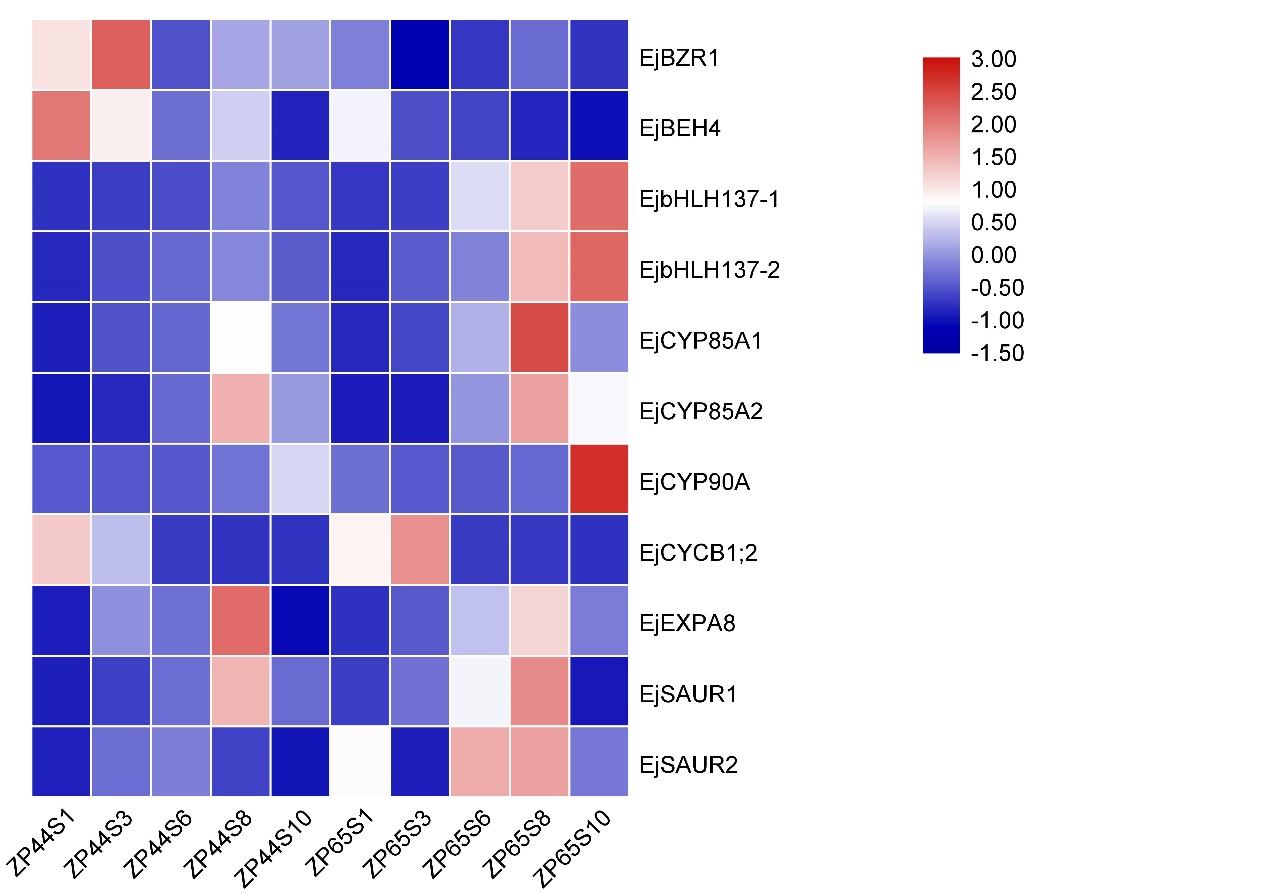
**

**Fig. S1** EjBZR1 related gene expression patterns based on transcriptome data of two breeding lines. S1, S3, S6, S8 and S10 were stages at 14, 42, 77, 104 and 133 days past anthesis of both ZP44 and ZP65 lines.

**
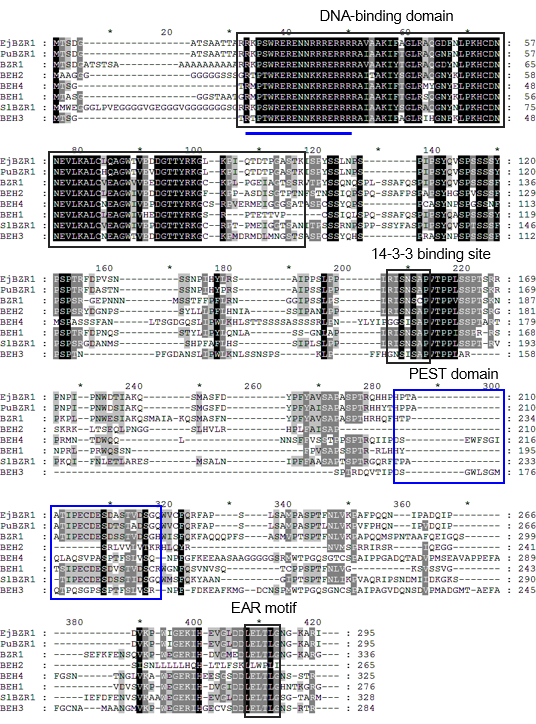
**

**Fig. S2** Protein sequence alignment and phylogenetic tree of EjBZR1s and other BES1/BZR1 homologues. Blue line indicated the nuclear localization signal sequence, black boxes mentioned the conserved DNA binding domain, 14-3-3 binding site and EAR motif, while the blue box indicated the PEST domain. Identical residues are shown in black and conserved residues in gray.

**
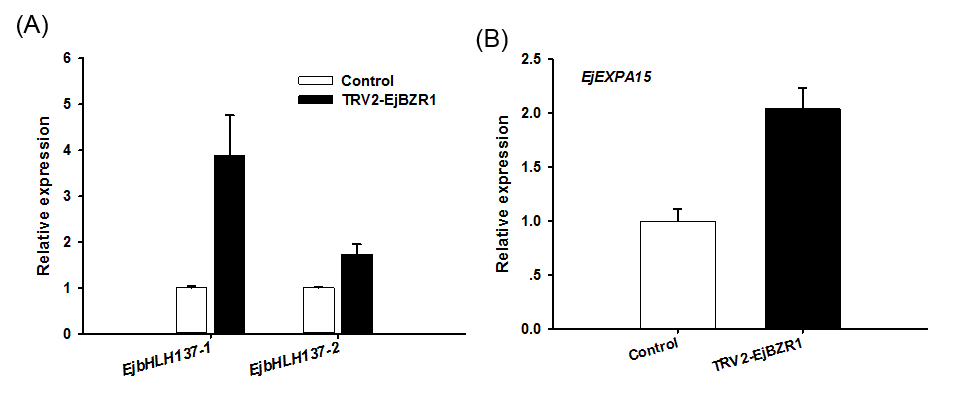
**

**Fig. S3** Gene expression of *EjBZR1* VIGS-treated loquat fruits comparing with the control fruits. (A) Reduction of *EjBZR11* by VIGS elevates *EjbHLH137*s expression levels. (B) Gene Reduction of *EjBZR11* by VIGS elevates *EjEXPA15*, the cell expansion-related gene, expression levels.


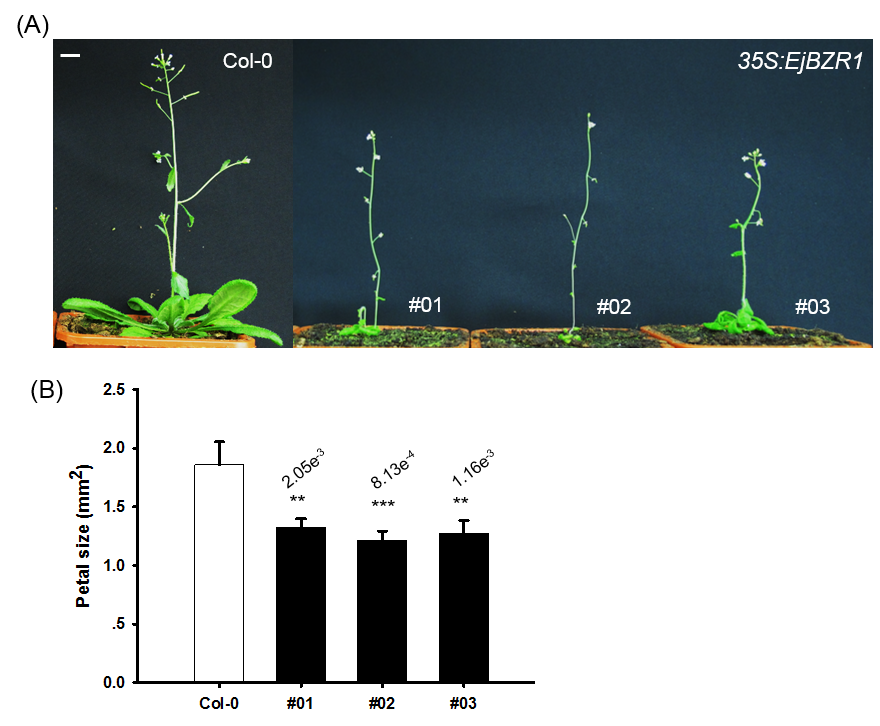


**Fig. S4** Overexpression of *EjBZR1*results into small plant size. (A) *EjBZR1* overexpression reduces plant height 2 months after germination in all OE lines. (B) *EjBZR1*overexpression significantly reduces petal size at bloom.

**
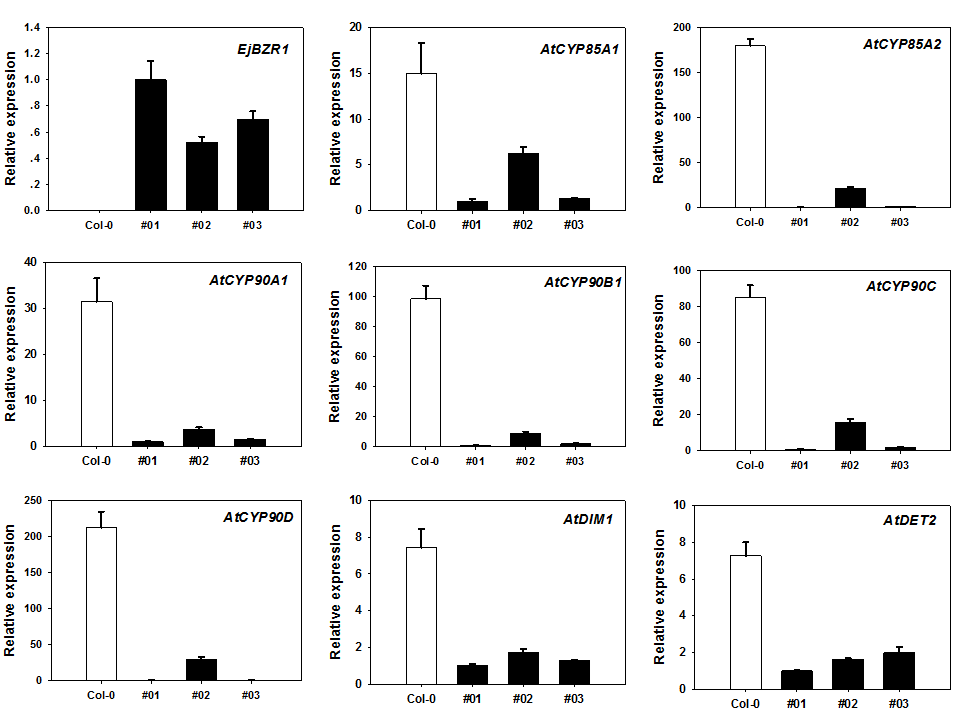
**

**Fig. S5** Gene expression in 4-week old plant of wild type and overexpression lines.

*EjBZR1*expression level greatly elevated in all OE lines and the BR biosynthesis-related genes expression levels were reduced.

**
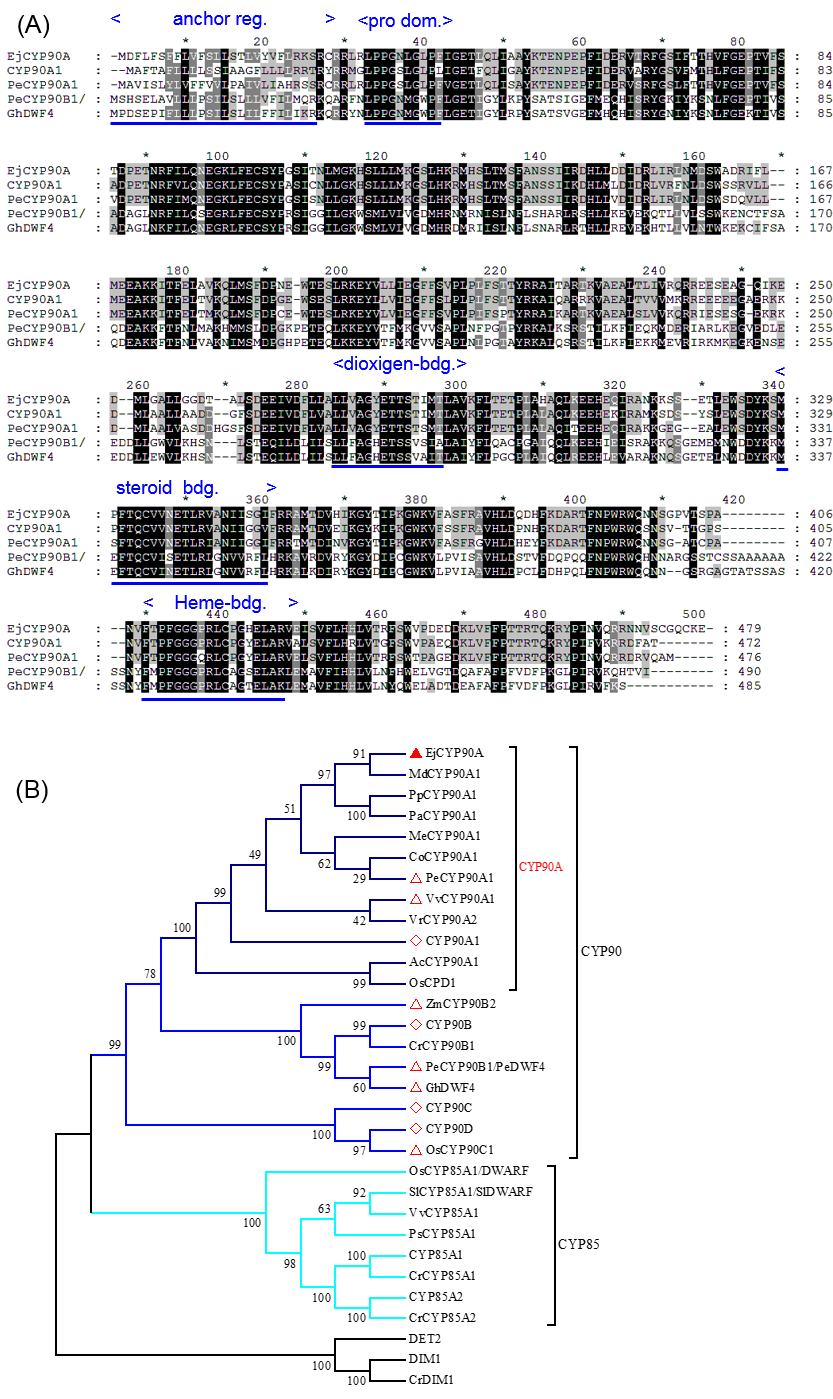
**

**Fig. S6** Multiple sequence alignment and phylogenetic analysis of amino acid sequences of EjCYP90A with other cytochrome P450s from different species. (a) Sequence alignments of EjCYP90A with other CYTOCHROME P450s. Amino acid sequences were aligned with the software of Genedoc. The EjCYP90A is characterized by N-terminal membrane-anchoring, proline-rich, oxygen- and heme- binding domains, which are highly conserved in most CYP450 proteins. Identical and similar amino acid residues are shaded with black and gray, respectively. (b) The phylogenetic relationship of EjCYP90A with other CYP450 proteins was constructed by the Neighbor-Joining method in MEGA6 software. The bootstrap values are shown as percentages at the nodes. The GenBank accession numbers of P450s as follows: AcCYP90A1 (*Ananas comosus*, OAY72352), CYP90A1 (*Arabidopsis thaliana*, NP196188), CYP90B (*Arabidopsis thaliana*, NP190635), CYP90C (*Arabidopsis thaliana*, NP568002), CYP90D (*Arabidopsis thaliana*, NP566462), DIM1 (*Arabidopsis thaliana*, NP001319595.1), DET2 (*Arabidopsis thaliana*, NP181340), CYP85A1 (*Arabidopsis thaliana*, NP851105), CYP85A2 (*Arabidopsis thaliana*, NP566852), CoCYP90A1 (*Corchorus olitorius*, OMO76031), CrCYP90B1 (*Capsella rubella*, XP006290928), CrCYP85A1 (*Capsella rubella*, XP006283674), CrCYP85A2 (*Capsella rubella*，XP006290963), CrDIM1 (*Capsella rubella*, XP006297345), GhDWF4 (*Gossypium hirsutum*, ABJ90340), MeCYP90A1 (*Manihot esculenta*, XP021618503), MdCYP90A1 (*Malus domestica*, XP008369248), OsCYP90C1 (*Oryza sativa*, BAB56089), OsCPD1 (*Oryza sativa*, BAG87383), OsCYP85A1/DWARF (*Oryza sativa*, BAC45000), PaCYP90A1 (*Prunus avium*, XP021805589), PeCYP90A1 (*Populus euphratica*, ADK66927), PeCYP90B1/ Pe DWF4 (*Populus euphratica*, AER08630), PpCYP90A1 (*Prunus persica*, XP007220390), PsCYP85A1 (*Pisum sativum*, BAF56235), SlCYP85A1/SlDWARF (*Solanum lycopersicum*, AAB17070), VrCYP90A2 (*Vigna radiata*, AAF89209), VvCYP85A1 (*Vitis vinifera*, ABB60086), VvCYP90A1 (*Vitis vinifera*, RVW81366), ZmCYP90B2 (*Zea mays*, PWZ56242).

**
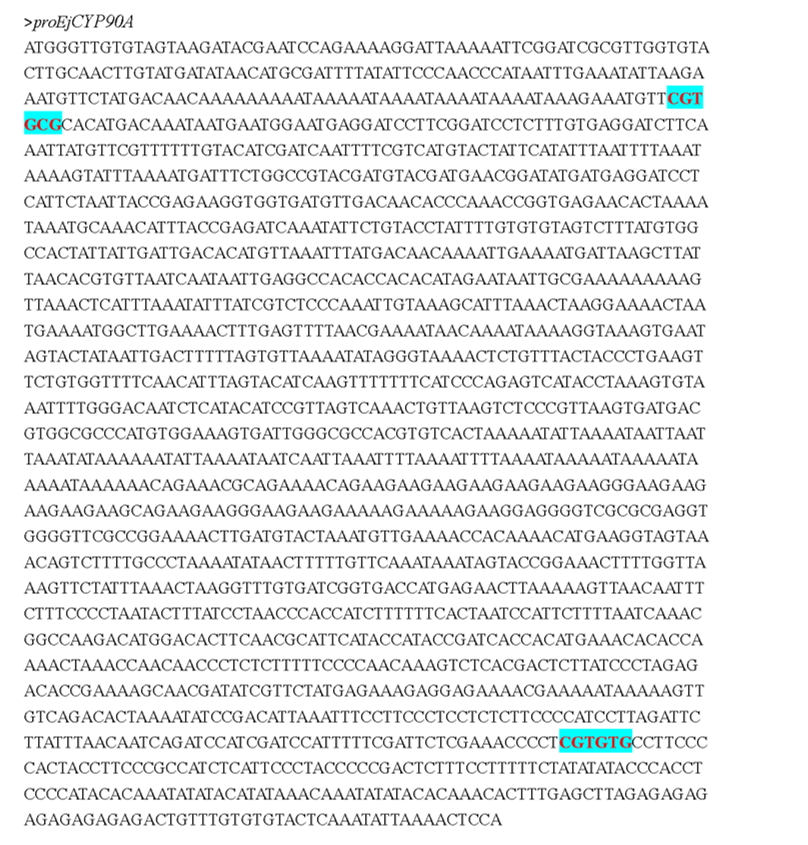
**

**Fig. S7** Promoter sequence of *EjCYP90A* with the BR response elements (CGTGT/CG) were in red and bolded font.

**Table S1** Correlations of fruit weight with diverse fruit traits in 13 loquat accessions.

| **Loquat Species** | | **Fruit weigth**  **(g)** | **Fruit diameter（mm）** | **Pericarp width**  **(mm)** | **Cell layer** | **Cell size**  **(10^3^ μm^2^)** |
| --- | --- | --- | --- | --- | --- | --- |
|  |  | **R** | 0.9545 | 0.9667 | 0.9007 | 0.9809 |
| **Cultivated species**  ***Eriobotrya japonica*** | ZP65 | 82.69±1.49 | 51.88±0.44 | 11.44±0.22 | 70.73±2.00 | 13.75±0.29 |
|  | Jiefangzhong | 60.37±0.88 | 46.38±0.28 | 8.53±0.09 | 56.70±2.31 | 9.95±1.03 |
|  | Zaozhong-6 | 40.12±0.63 | 40.58±0.19 | 7.21±0.21 | 61.86±1.92 | 7.80±0.45 |
|  | ZP44 | 15.21±0.37 | 29.26±0.25 | 4.92±0.15 | 51.33±1.15 | 4.69±0.05 |
|  | Puye | 7.04±0.18 | 24.12±0.22 | 3.42±0.07 | 32.90±1.20 | 3.19±0.35 |
| **Wild species**  ***Eriobotrya* genus** | *E. serrate* | 9.73±1.23 | 22.20±1.28 | 5.12±0.38 | 39.70±2.63 | 5.91±0.66 |
|  | *E. daduheensis* | 2.74±0.14 | 14.83±0.27 | 2.80±0.10 | 30.50±1.43 | 2.81±0.15 |
|  | *E. deflexa* | 2.39±0.23 | 17.26±0.69 | 2.70±0.13 | 34.40±2.55 | 2.88±0.09 |
|  | *E. prinoides* var*.laotica* | 1.57±0.08 | 14.40±0.33 | 2.84±0.09 | 23.10±2.08 | 3.28±0.07 |
|  | *E. salwinensis* | 1.45±0.42 | 13.76±0.79 | 2.29±0.32 | 28.85±2.85 | 2.53±0.25 |
|  | *E. deflexa* f. *koshunensis* | 1.35±0.51 | 12.79±1.43 | 1.76±0.22 | 29.30±2.11 | 2.60±0.25 |
|  | *E. prinoides* | 0.73±0.01 | 10.33±0.11 | 1.98±0.06 | 20.10±1.91 | 2.28±0.26 |
|  | *E. henryi* | 0.53±0.07 | 8.64±0.63 | 0.87±0.17 | 19.90±1.37 | 1.88±0.14 |

**Table S2** Primers used for vector construction.

| Primer applications | Primer name | Sequence imformation（5′→3′） |
| --- | --- | --- |
| Overexpression | 35S::EjBZR1-F | acgggggactctaga**ggatcc** (*BamH* I) ATGACGTCTGATGGGGC |
|  | 35S::EjBZR1-R | cgatcggggaaattc**gagctc**(*Sac* I) TTAAATCCGAGCCTTTCCATTC |
|  | 35S:: EjCYP90A-F | acgggggactctaga**ggatcc** (*BamH* I)ATGGATTTCCTCTTCTCG |
|  | 35S:: EjCYP90A-R | cgatcggggaaattc**gagctc**(*Sac* I) TTACTCTTTACATTGCCCAC |
| Subcellular  localization | EjBZR1-GFP-F | ataagcttgatatc**gaattc**(*EcoR* I) ATGACGTCTGATGGGGC |
|  | EjBZR1-GFP-R | tttactcatactagt**ggatcc** (*BamH* I)AATCCGAGCCTTTCCATTC |
| VIGS | TRV2-EjBZR1-F | gtgagtaaggttacc**gaattc**(*Xho* I)AGGAAGCCGTCGTGG |
|  | TRV2-EjBZR1-R | tgtcttcgggacatg**cccggg**(*Xma* I)TGGGGCGTCTGGAG |
|  | TRV2-EjCYP90A -F | gtgagtaaggttacc**gaattc**(*Xho* I)TGCCTCCGGGGAATC |
|  | TRV2-EjCYP90A -F | tgtcttcgggacatg**cccggg**(*Xma* I) TGGCCTCCTCCATGAG |
| Dual-luciferase  assays | 62SK-EjBZR1-F | cgctctagaactagt**ggatcc**(*BamH* I)ATGACGTCTGATGGGGC |
|  | 62SK-EjBZR1-R | gtcgacggtatcgat**aagctt**(*Hind* III)TTAAATCCGAGCCTTTCCATTC |
|  | proEjCYP90-0800-F | gtcgacggtatcgat**aagctt**(*Hind* III)ATGGGTTGTGTAGTAAGATACG |
|  | proEjCYP90-0800-R | cgctctagaactagt**ggatcc**(*BamH* I)TGGAGTTTTAATATTTGAGTACACAC |
| Y1H | pAbAi-CYP90-F | gaaaagcttgaattc**gagctc**(*Sac* I) ACCCCTCGTGTGCCTTCC |
|  | pAbAi-CYP90-R | atacagagcacatgc**ctcgag**(*Xho* I)GGAAGGCACACGAGGGGT |
|  | pGADT7-BZR1-F | gccatggaggccagtgaattc (*EcoR* I)ATGACGTCTGATGGGGC |
|  | pGADT7-BZR1-R | acgattcatctgcagctcgag (*Xho* I)TTAAATCCGAGCCTTTCCATTC |

**Table S3** Primers used for gene expression analyses.

| Primer name | Forward primer (5'-3') | Reverse primer (5'-3') |
| --- | --- | --- |
| qEjBZR1 | TACCCGTTTTATGCCGTCTC | GGAGCAAATCTCTGGAAGCA |
| qEjCYP85A1 | GAAAGGAGCTGGGGATAACA | CATTCGGGGCCTCTACTCT |
| qEjCYP85A2 | GGAAAGGAGCTGGGAATATCA | CCATTCGGTGCCTCTACTCT |
| qEjCYP90A | AGACCAATCGGTTCATCCTG | GTTTGCAAAGCTCATGGTCA |
| qEjRPL18 | ATGGGATTTGGCTTCGTTATC | AGAGTT TTGCTGGGATGGTG |
| qAtUBQ10 | GGACCAGCAGCGTCTCATCTTCGCT | CTTATTCATCAGGGATTATACAAG |
| qAtCYP85A1 | GGACGTGAAGTCAATGAAGTTCACT | TTCCTTACCAGGACAAAGCCTTGTC |
| qAtCYP85A2 | CGAACCGCTCACTCTCGACGAT | AATAGCTCTTTGATTCTAAGCTCT |
| qAtCYP90A1 | GAATGGAGTGATTACAAGTC | GTGAACACATTAGAAGGGCCTG |
| qAtCYP90B | GAAGGAACTAGGAGAGTCAG | CCACGTCGAAAAACTACCACTTC |
| qAtCYP90C1 | GGAGATGAAGAGGCGTAAATTGGA | GCAAATACTGCTGTTTGCCGATCC |
| qAtCYP90D1 | GTCAAATTCCTCTCTGATTCTCCTG | TCGAGACCAGGGCACAATCTCTGAC |
| qAtDIM1 | CTCGAATGGGTCCACCGCGAAATG | CATACAATTCACCATTAAACATTC |
| qAtDET2 | AATCTCCTCAATGGTTATATC | CGTGTACAGAAAAAATCCAATACC |
